# Effect of bariatric surgery on glycemic and metabolic outcomes in people with obesity and type 2 diabetes mellitus: a systematic review, meta-analysis, and meta-evidence of 39 studies

**DOI:** 10.3389/fnut.2025.1603670

**Published:** 2025-06-23

**Authors:** Xiao Wei, Haijuan Yuan, Daorong Wang, Jing Zhao, Fang Fang

**Affiliations:** ^1^Department of Gastrointestinal Surgery, Northern Jiangsu People’s Hospital Affliated to Yangzhou University/Clinical Medical College, Yangzhou University, Yangzhou, China; ^2^Department of Nursing, Northern Jiangsu People’s Hospital Affliated to Yangzhou University/Clinical Medical College, Yangzhou University, Yangzhou, China

**Keywords:** bariatric surgery, diabetes mellitus, meta-analysis, obesity, systematic review

## Abstract

**Background:**

Bariatric surgery has become a widely utilized therapeutic approach for obesity management and glycemic regulation in individuals with type 2 diabetes mellitus (T2DM). This meta-analysis examines the effects of bariatric surgery on key glycemic and metabolic parameters.

**Methods:**

A systematic literature search was performed across PubMed, Scopus, Embase, and Web of Science to identify relevant studies assessing alterations in outcomes following bariatric surgery compared to baseline measurements. Eligible studies were analyzed using a random-effects model to compute weighted mean differences (WMD) and their corresponding 95% confidence intervals (CIs).

**Results:**

Bariatric surgery resulted in 39 with 3,855 participants in significant reductions in fasting blood glucose (FBG) (WMD: −0.82 mg/dL; 95%CI: −0.92 to −0.72), postprandial glucose (PPG) (WMD: −4.15 mg/dL; 95%CI: −5.38 to −2.92), Homeostatic Model Assessment for Insulin Resistance (HOMA-IR) levels (WMD: -2.81; 95% CI: −3.06 to −2.56), C-peptide (WMD: -0.38; 95%CI: −0.73 to −0.03) and fasting insulin (WMD: -0.62; 95% CI: −0.88 to −0.36). No significant reduction in glycated hemoglobin (HbA1c) levels was observed (WMD: -0.17; 95%CI: −0.39 to 0.04). Follow-up periods ranging from 2.3 to 120 months.

**Conclusion:**

It was concluded that the bariatric surgery may have improved the glycemic and metabolic outcomes. Therefore, the results underscore the value of incorporating bariatric surgery into diabetes care strategies, highlighting its potential to enhance long-term diabetes management and mitigate the risk of complications.

## Introduction

Globally, obesity and type 2 diabetes mellitus (T2DM) have reached epidemic proportions. According to recent WHO data, one in eight people worldwide was living with obesity in 2022, and nearly half of all adults (43%) are overweight.^1^ Concurrently, the prevalence of diabetes has surged: by 2022 an estimated 830 million people had diabetes worldwide.^2^ Obesity and T2DM are tightly linked through complex metabolic and inflammatory pathways. Expanded adipose tissue in obesity secretes dysregulated adipokines and cytokines, leading to chronic low-grade inflammation. Adipocyte hypertrophy and hypoxia provoke macrophage infiltration into adipose tissue and release of factors such as pro-inflammatory cytokines. These pro-inflammatory signals impair insulin receptor signaling in muscle and liver, reducing glucose uptake and increasing hepatic gluconeogenesis, while also promoting pancreatic *β*-cell dysfunction (1).

Bariatric surgery has emerged as the most effective treatment for severe obesity, consistently producing greater and more durable weight loss than non-surgical interventions. In addition to weight loss, bariatric procedures confer profound metabolic benefits. Roux-en-Y gastric bypass (RYGB) and sleeve gastrectomy (SG) in particular achieve sustained weight reduction beyond 10 years while dramatically improving obesity-related comorbidities (2). These weight-independent effects implicate altered gut physiology as key mechanisms. Postoperatively, patients typically show exaggerated postprandial incretin responses: elevations in glucagon-like peptide-1 (GLP-1) and peptide YY (PYY) that enhance insulin secretion and satiety (3, 4). RYGB, by bypassing the proximal intestine, acutely delivers nutrients to the distal gut and evokes strong GLP-1 and bile acid signals, whereas SG reduces ghrelin (a hunger hormone) and similarly boosts GLP-1/PYY responses (5). In parallel, adipose-derived inflammation is attenuated after surgery (6).

Despite clear overall benefits, debate persists regarding which bariatric procedure optimally treats T2DM. Observational and trial evidence often favors RYGB for glycemic remission. For example, in a pooled analysis of randomized trials, RYGB produced a higher diabetes remission rate than SG at 1 year (7). However, differences tend to attenuate over time: in studies with 2–5-year follow-up, remission rates with SG approached those of RYGB (5, 7). Adjustable gastric banding (AGB) is generally less effective. Comparative data show much lower remission rates after AGB than after RYGB or SG (8). For instance, a landmark U. K. cohort found 2-year remission in only ~7% of AGB patients versus ~41% after RYGB and 26% after SG (8). Biliopancreatic diversion (BPD) or duodenal switch, a more complex procedure, yields the highest remission (often >90%) (8). A recent study of insulin-treated patients confirmed that BPD-DS conferred superior long-term remission compared to other procedures (9). The literature contains some conflicting signals: some reviews assert RYGB’s superiority, while others find only transient differences (5, 7). Variability among patients (age, diabetes duration, baseline *β*-cell function, etc.) also affects outcomes. For example, the one study found that patients with lower preoperative hemoglobin A1c (HbA1c) and fewer diabetes medications were far more likely to maintain remission at 10 years (10).

Given these gaps, a comprehensive synthesis of the most recent evidence is needed. Our systematic review and meta-analysis evaluates all major bariatric procedures (RYGB, SG, AGB, and BPD) and their effects on a wide range of glycemic (HbA1c, fasting blood glucose (FBG), postprandial glucose (PPG)) and metabolic markers (fasting insulin, homeostatic model assessment of insulin resistance (HOMA-IR), C-peptide) in people with T2DM. This broad comparison offers a more complete picture of their relative efficacy. Importantly, we formally assess the certainty of the evidence using GRADE methodology to provide clinicians with clearer guidance.

## Methods

This study was conducted in accordance with the Preferred Reporting Items for Systematic Reviews and Meta-Analyses (PRISMA) guidelines (11). It was registered in PROSPERO with an ID: CRD420251063420.

### Search strategy

A thorough literature search was conducted across several electronic databases, including PubMed, EMBASE, Scopus, and Web of Science, from their inception until November 2024, without language restrictions. Clinical trial registries such as ClinicalTrials.gov and the WHO International Clinical Trials Registry Platform (ICTRP)—were also screened for ongoing or unpublished studies to minimize publication bias. The search strategy incorporated MeSH terms and keywords related to bariatric surgery, type 2 diabetes mellitus, and metabolic outcomes.

### Eligibility criteria

All identified citations were imported into EndNote X9 (Thomson Reuters, New York) for reference management, with duplicate records subsequently eliminated. Two independent investigators screened the titles and abstracts of the remaining publications to determine eligibility. Eligible studies underwent full-text review, during which they were assessed for compliance with the predefined inclusion and exclusion criteria. The inclusion criteria were: Population: Adults (≥18 years) with a confirmed diagnosis of T2DM and obesity (BMI ≥ 30 kg/m^2^). Exposure and Comparator: Any form of bariatric surgery, including RYGB, SG, AGB, or BPD compared with pre-surgery baseline data. Outcomes: Changes in HbA1c, FBG, postprandial glucose, C-peptide levels, HOMA-IR, and fasting insulin levels. Study Design: Randomized controlled trials (RCTs), cohort studies, or quasi-experimental studies with sufficient data for quantitative synthesis. Exclusion criteria included studies involving patients with type 1 diabetes (T1D); those without pre- and post-operative blood glucose data; Case reports, conference abstracts, and non-English-language publications.

### Research selection

Studies were independently screened and selected by two authors based on inclusion criteria, with agreement reached after consultation with the third reviewer. These authors were also responsible for extracting relevant data, including the first author’s name, year of publication, sample size, study design, Mean age, sex distribution, BMI, diabetes duration, and baseline glycemic/metabolic values and outcome data.

### Study risk of bias assessment and meta-evidence

Methodological quality assessment was performed using the Cochrane Risk of Bias 2.0 (RoB 2) tool for RCTs (12), evaluating key domains including randomization processes, deviations from intended interventions, and outcome reporting. For non-randomized trials, the Risk of Bias in Non-Randomized Studies of Interventions (ROBINS-I) tool was applied to assess potential biases arising from confounding, participant selection, and outcome measurement (13). Two independent reviewers conducted the assessments, with any discrepancies resolved through consensus discussion. The overall certainty of evidence was subsequently appraised using the GRADE (Grading of Recommendations, Assessment, Development, and Evaluation) framework (14).

### Statistical analysis

The data were analyzed using the STATA version 17 software. The weighted mean difference (WMD) or standardized mean difference (SMD) with 95% confidence intervals (CI) was described for continuous outcomes. Since all included trials were heterogeneous by design, the data were analyzed using a random effects model. Heterogeneity across RCTs was assessed using prediction intervals instead of the traditional *I*^2^ statistic (15). Between-study heterogeneity was quantified using *I*^2^ statistics and τ^2^ estimates. A leave-one-out sensitivity analysis was carried out to examine whether any individual study influenced the overall effect size estimates (16). Publication bias was assessed via funnel plot symmetry and Egger’s regression test (17).

## Results

### Search results

**Figure 1:**
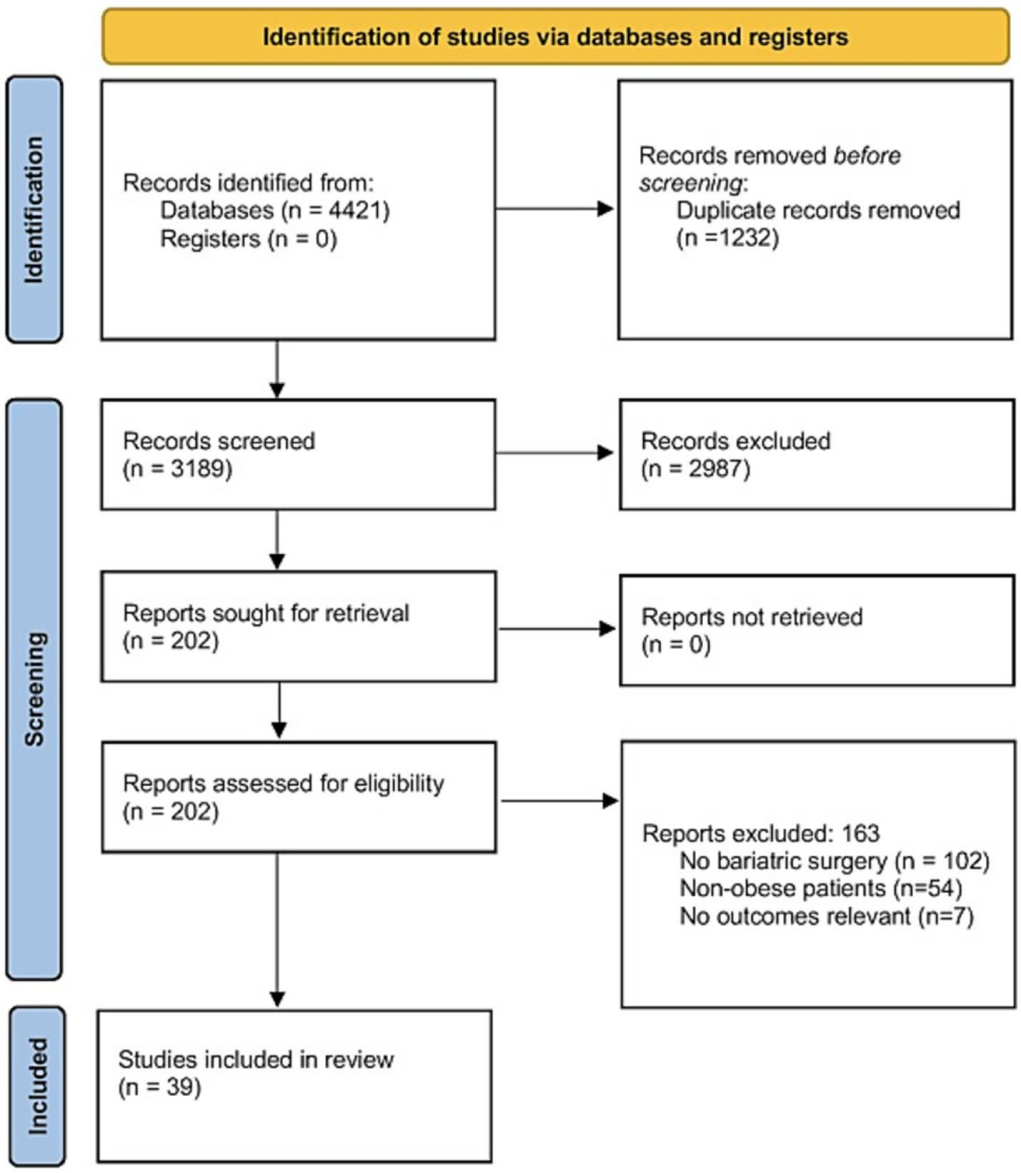
Flow diagram of study selection.

A total of 4,421 studies were identified through the literature search and subsequently screened. After removing duplicates, 3,189 records were screened based on titles and abstracts, resulting in 202 full-text articles for further evaluation. 163 full-text articles were then excluded, due to the following reasons: no bariatric surgery (*n* = 102), non-obese patients (*n* = 54), and non-relevant outcomes (*n* = 7). Finally, a total of 39 eligible studies met all inclusion criteria (18–56). The PRISMA flow diagram is depicted in Figure 1.

### Characteristics of the included studies

A total of 39 studies were included in this meta-analysis, spanning a diverse range of global settings. The majority of studies originated from Asia, the Americas, and Europe, with China, Korea, Brazil, and the United States being the most frequently represented countries. This reflects the broad geographic distribution of bariatric surgical research. Sample sizes across studies varied from 6 to 479 participants, with mean ages ranging between 41.3 and 63.8 years. The studies evaluated various surgical interventions, including RYGB (Roux-en-Y Gastric Bypass), LAGB (Laparoscopic Adjustable Gastric Banding), and DJB (Duodenal-Jejunal Bypass), with follow-up periods ranging from 2.3 to 120 months. Risk of bias assessments varied across studies, with 15 classified as “High,” 15 as “Moderate” or having “Some concerns,” and 9 as “Low” (Table 1). Considering the GRADE quality of evidence, FBG, PPG, C-peptide levels, HOMA-IR, and fasting insulin levels had high GRADE quality. Low quality was noted for HbA1c.

**Table 1:** Characteristics of the included studies.

Author and year of publication	Country	Study design	Sample size	Gender ratio (M/F)	Type of surgery	Mean age (in years)	Follow-up period (months)	Diabetes duration (in years)	Risk of bias
Ramos et al. (18)	Brazil	Prospective	20	11/9	DJB	43	6	5.3	Moderate
Depaula et al. (19)	Brazil	Prospective	69	47/22	LII + DSG	51 ± 5.6	21.7	11 ± 4	Moderate
Geloneze et al. (20)	Brazil	Prospective	12	9/3	DJB	50 ± 5.3	6	9 ± 2	High
Kim et al. (21)	Korea	Prospective	10	2/8	LMGB	49.6	6	6.6	Low
Lee et al. (22)	Korea	Prospective	6	6/0	DJB	50.2	6	5.5	High
Navarrete et al. (23)	Venezuela	Prospective	10	5/5	LSG + DJB	46.5	12	<10	High
Scopinaro et al. (24)	Italy	Prospective	15	13/2	BPD	57.8 ± 6.7	24	11.1 ± 6.1	Moderate
Dixon et al. (25)	Korea	Prospective	103	41/62	LMGB+RYGB	47.7 ± 9.6	12	8.2 ± 5	High
Shrestha et al. (27)	China	Prospective	33	24/9	RYGB	49.5 ± 1.3	3	<10	Moderate
García et al. (26)	Spain	Prospective	13	10/3	BAGUA	63.8 ± 8.3	6	16.9 ± 8.7	High
Blanco et al. (28)	Spain	Prospective	7	NR	RYGB	NR	24	NR	High
Maraka et al. (29)	United States	Prospective	118	NR	RYGB	55.0 ± 10.1	24	NR	Moderate
Robert et al. (30)	Canada	Prospective	20	NR	BPD or SG	40.9 ± 4.2	55.1	NR	Moderate
Chen et al. (31)	China	Retrospective	35	22/13	RYGB	45.3 ± 8.5	12	3.7 ± 2.4	High
Cui et al. (32)	China	Retrospective	58	36/22	RYGB	48.5 ± 12.3	12	< 15	High
Di et al. (33)	China	Retrospective	66	28/NR	RYGB	50.4 ± 11.4	36	8.9 ± 5.2	Moderate
Gong et al. (34)	China	Prospective	31	14/17	RYGB	46.2 ± 11.1	6	8.3 ± 5.7	Moderate
Heo et al. (35)	Korea	Prospective	31	19/12	DJB	46.6 ± 7.7	12	8.3 ± 4.7	Low
Ke et al. (36)	China	Retrospective	47	26/21	RYGB	47.45 ± 8.69	24	5.58 ± 4.40	Moderate
Kim et al. (37)	Korea	Prospective	172	NR	SAGB	46 ± 11	36	9.6 ± 5.2	Low
Lee et al. (38)	Taiwan	Prospective	80	30/50	GB/SG	47.7 ± 9.1	12	6.5 ± 5.1	Moderate
Liang et al. (39)	China	Prospective	80	37/43	RYGB	48.52	12	7	Low
Malapan et al. (40)	Taiwan	Prospective	29	13/16	RYGB	53	12	10.4	High
Wang et al. (41)	China	Retrospective	40	25/15	RYGB	49.13 ± 8.15	24	5.82 ± 2.85	High
Yin et al. (42)	China	Retrospective	28	8/20	RYGB	51.6	12	9.6	Moderate
Horwitz et al. (43)	America	RCT	57	NR	RYGB, LSG or LAGB	NR	36	NR	High
Hsu et al. (44)	China	Retrospective	52	11/41	LII-DSG	44.2	60	NR	High
Bhandari et al. (45)	India	Prospective	30	15/15	RYGB	41.3	12	3.86	Low
Wentworth et al. (46)	Australia	RCT	51	15/36	LAGB	53	24	2.5	High
Özmen et al. (47)	Turkey	Observational	244	NR	LSG and LSAGB	48.6	10.2	NR	Moderate
Adams et al. (48)	USA	RCT	420	337/83	GBP, gastric bypass	43.4	2.3	NR	High
Serrot et al. (49)	USA	RCT	17	13/4	RYGB	56	12	NR	High
Leonetti et al. (50)	Italy	Prospective	60	19/41	LII-DSG	NR	18	NR	Moderate
Pories et al. (51)	USA	Prospective	479	NR	GGB	NR	120	NR	Low
Dixon and O’Brien (52)	Australia	Prospective	50	17/33	LAGB	NR	12	NR	Low
Brancatisano et al. (53)	Australia	Prospective	78	33/45	LAGB	52	12.5	5	Moderate
Schauer et al. (54)	USA	RCT	150	51/99	GB & LII-DSG	49	60	8.4	Low
O’Brien et al. (55)	Australia	RCT	40	10/30	LAGB	41.8	24	NR	Low
Hofsø et al. (56)	Norway	RCT	76	NR	RYGB	42.8	12	1	Some concerns

### FBG levels

**Figure 2:**
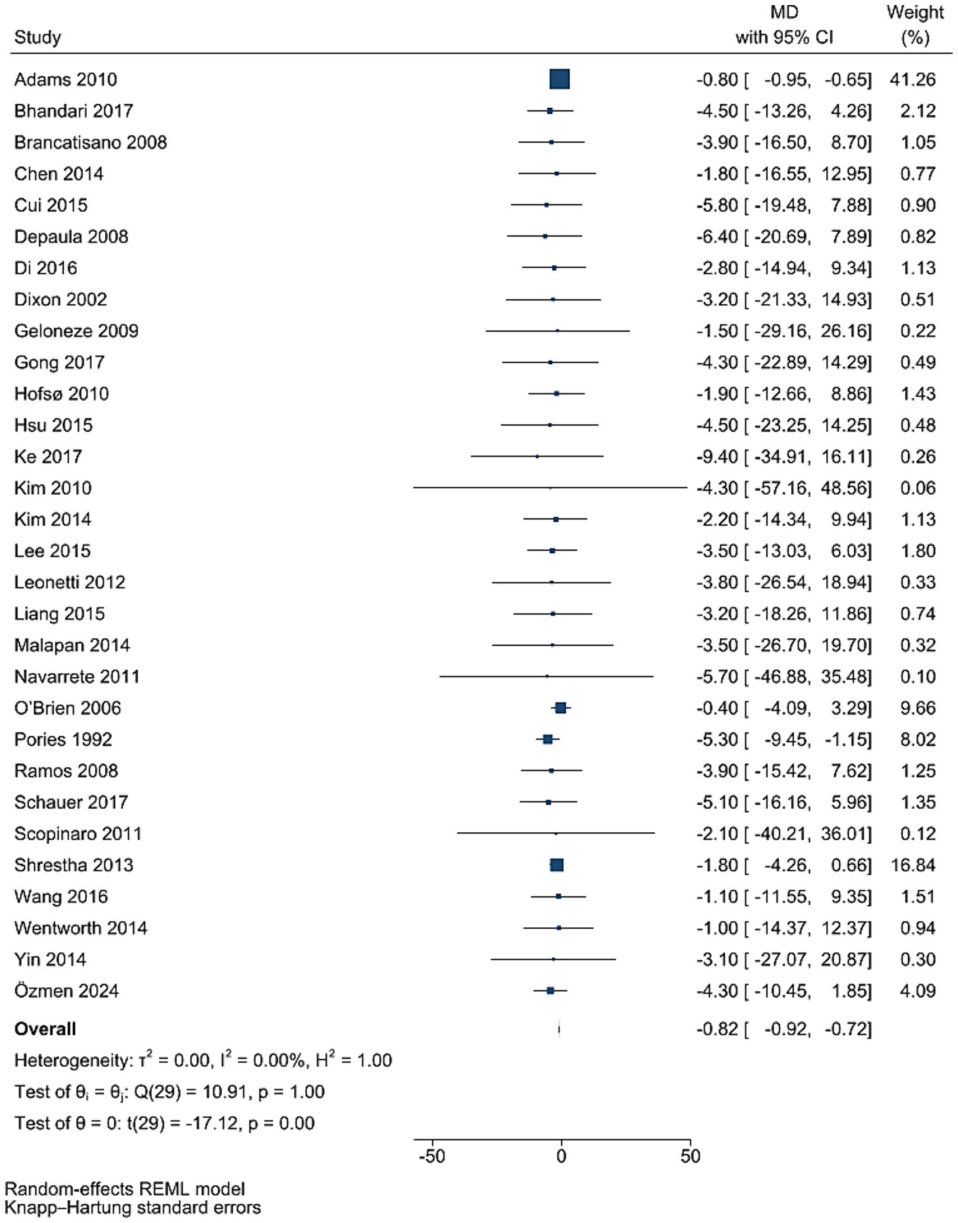
Forest plot detailing mean difference and 95% confidence intervals (CIs), the effects of bariatric surgery supplementation on FPG.

Data from 30 studies involving 3,494 participants showed that FBG levels decreased significantly after bariatric surgery compared to pre-surgery values (WMD = −0.82 mg/dL, 95% CI: −0.92, −0.72; *p* < 0.001, *I*^2^ = 0.0%; tau^2^ value 0.001; Figure 2). Further analysis based on sample size and mean age showed more reductions in studies with more than 50 individuals, and age less than 50 years, with age and sample size identified as a source of heterogeneity. Sensitivity analysis confirmed the robustness of the overall findings with WMD. Funnel plot was asymmetrical (Supplementary Figure S1) with highly significant Egger’s test (*p* = 0.02). Then, trim and fill analysis was performed with 45 studies (15 imputed studies, WMD = −0.81, 95% CI, −0.96, −0.65; *p* < 0.05; Supplementary Figure S2).

### HbA1C levels

**Figure 3:**
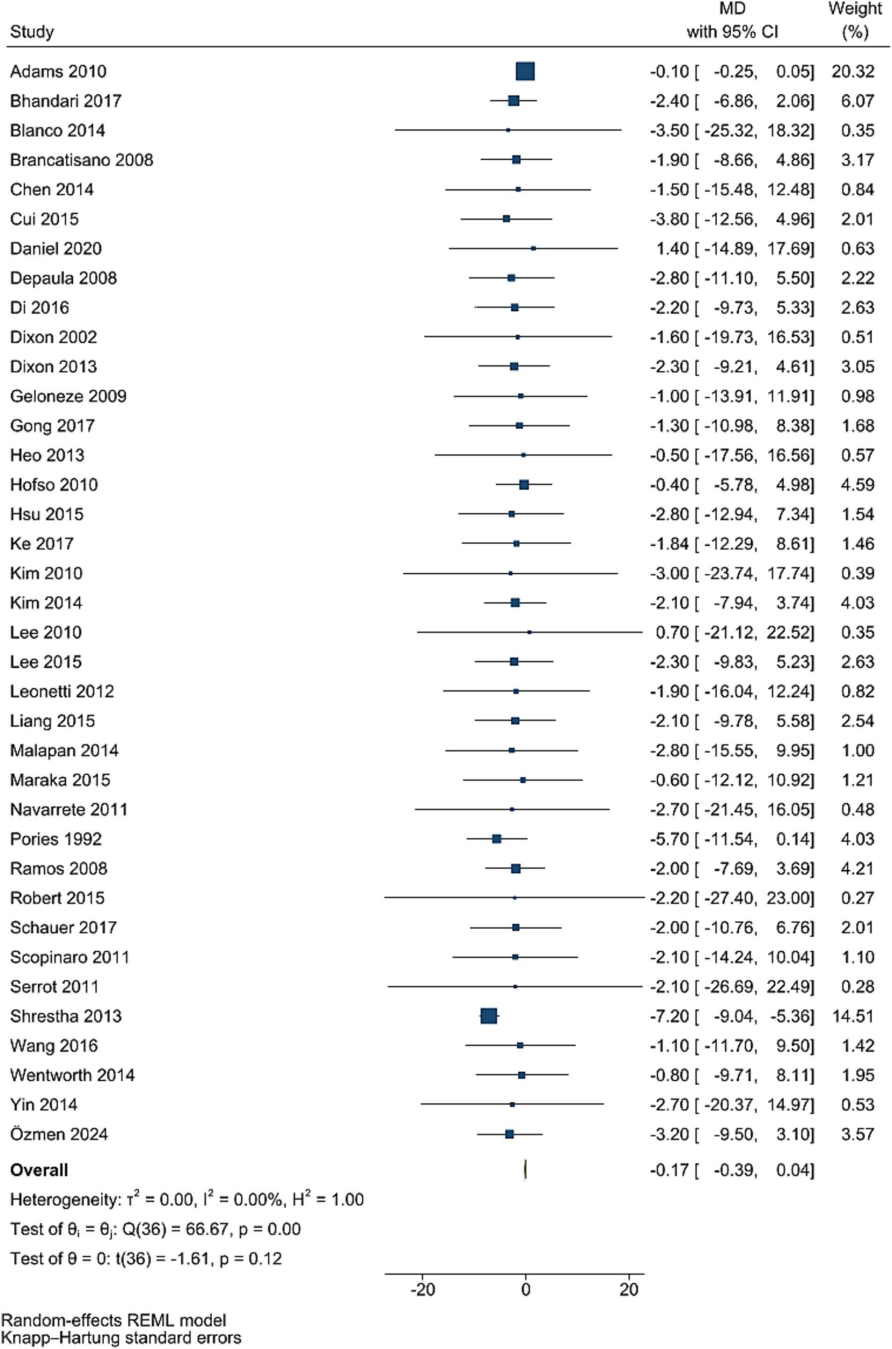
Forest plot detailing mean difference and 95% confidence intervals (CIs), the effects of bariatric surgery supplementation on HbA1C.

Data from 37 studies involving 3,855 participants showed that HbA1c levels did not significantly decrease after bariatric surgery compared to pre-surgery values (WMD = −0.17, 95% CI: −0.39, 0.04; *p* = 0.115, *I*^2^ = 0.0%; tau^2^ value 0.001; Figure 3). Further analysis based on sample size and mean age showed more reductions in studies with less than 50 individuals, and age less than 50 years, with age and sample size identified as a source of heterogeneity. Sensitivity analysis revealed no single RCT effect on the overall estimates. Funnel plot was asymmetrical (Supplementary Figure S3) with highly significant Egger’s test (*p* < 0.001). Then, trim and fill analysis was performed with 54 studies, and led to significant effect on HbA1c (17 imputed studies, WMD = −3.99, 95% CI: −5.21, −2.77; *p* < 0.05; Supplementary Figure S4).

### PPG levels

**Figure 4:**
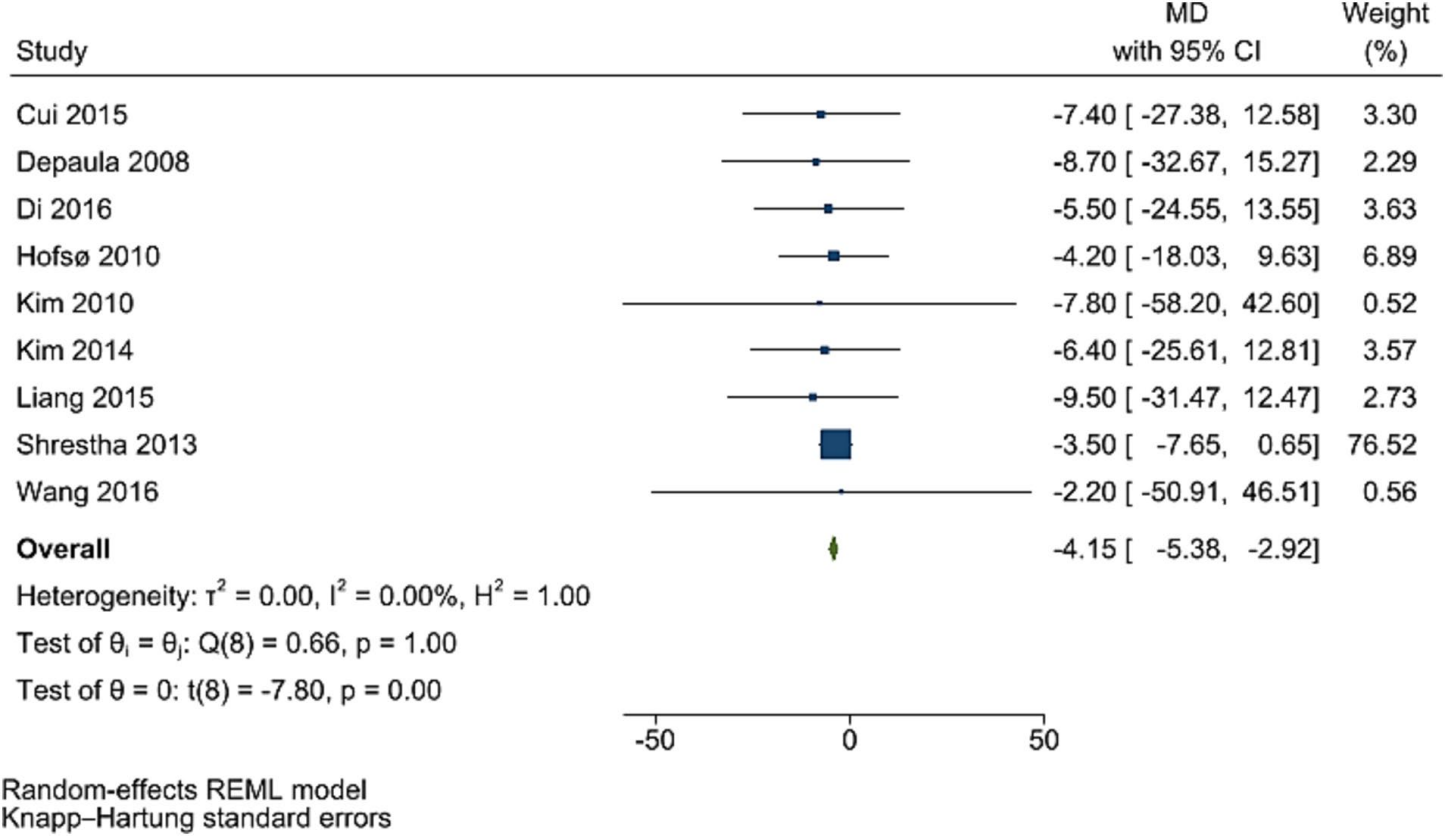
Forest plot detailing mean difference and 95% confidence intervals (CIs), the effects of bariatric surgery supplementation on PPG.

Post bariatric surgery, compared to pre-surgery values from nine studies involving 1,154 participants, led to a significant reduction in PPG levels (WMD = −4.15 mg/dL, 95% CI: −5.38, −2.92; *p* < 0.001, *I*^2^ = 0.0%; tau^2^ value 0.001; Figure 4). Sensitivity analyses revealed robust pooled estimates, with no single study significantly affecting the pooled effect size. Funnel plot was asymmetrical (Supplementary Figure S5) with highly significant Egger’s test (*p* = 0.532). Then, trim and fill analysis was performed with 13 studies (four imputed studies, WMD = −3.74, 95% CI: −7.22, −0.26; *p* < 0.05; Supplementary Figure S6).

### C-peptide levels

**Figure 5:**
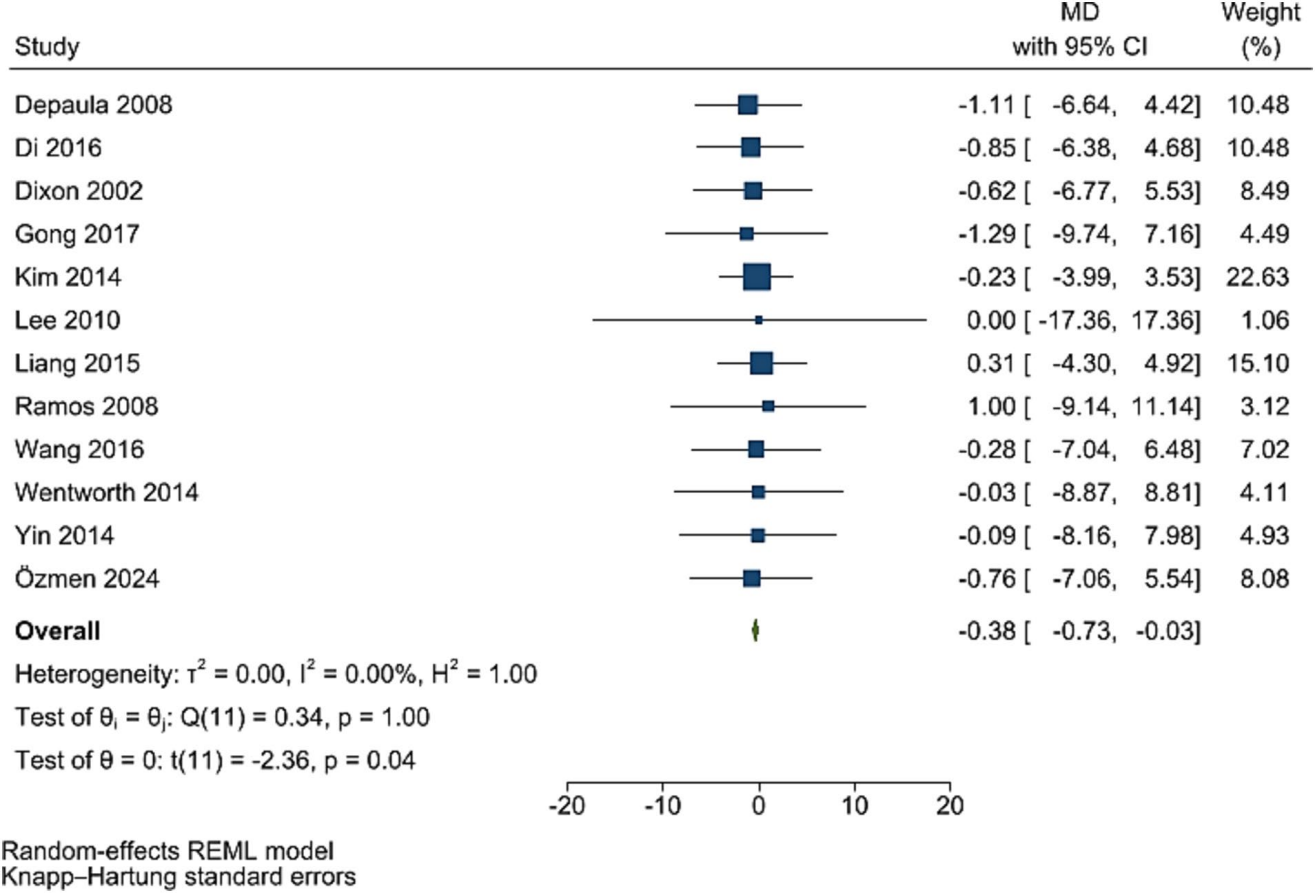
Forest plot detailing mean difference and 95% confidence intervals (CIs), the effects of bariatric surgery supplementation on c-peptide.

The improving effect of post-bariatric surgery compared to pre-surgery values on C peptide levels was significant (WMD = −0.38, 95% CI: −0.73, −0.03, *p* = 0.037, *I*^2^ = 0.0%; tau^2^ value 0.001, 12 studies involving 1,216 participants; Figure 5). Sensitivity analyses revealed robust pooled estimates. Egger’s test showed no evidence of publication bias (*p* = 0.988).

### HOMA-IR

**Figure 6:**
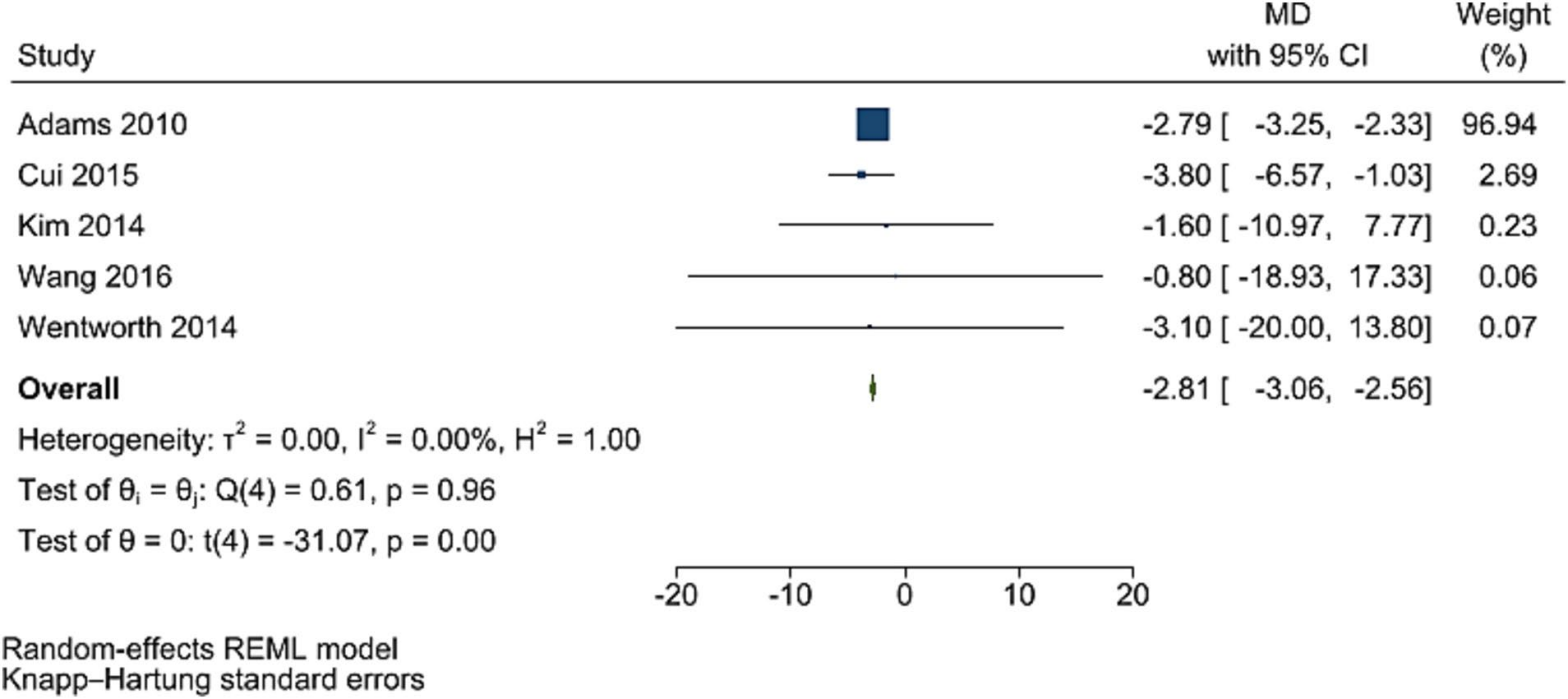
Forest plot detailing mean difference and 95% confidence intervals (CIs), the effects of bariatric surgery supplementation on HOMA-IR.

The improving effect of post-bariatric surgery compared to pre-surgery values on HOMA-IR levels was significant (WMD = −2.81, 95% CI: −3.06, −2.56, *p* < 0.001, *I*^2^ = 0.0%; tau^2^ value 0.001, five studies involving 1,361 participants; Figure 6). Sensitivity analyses revealed robust overall effect size estimates.

### Fasting insulin levels

**Figure 7:**
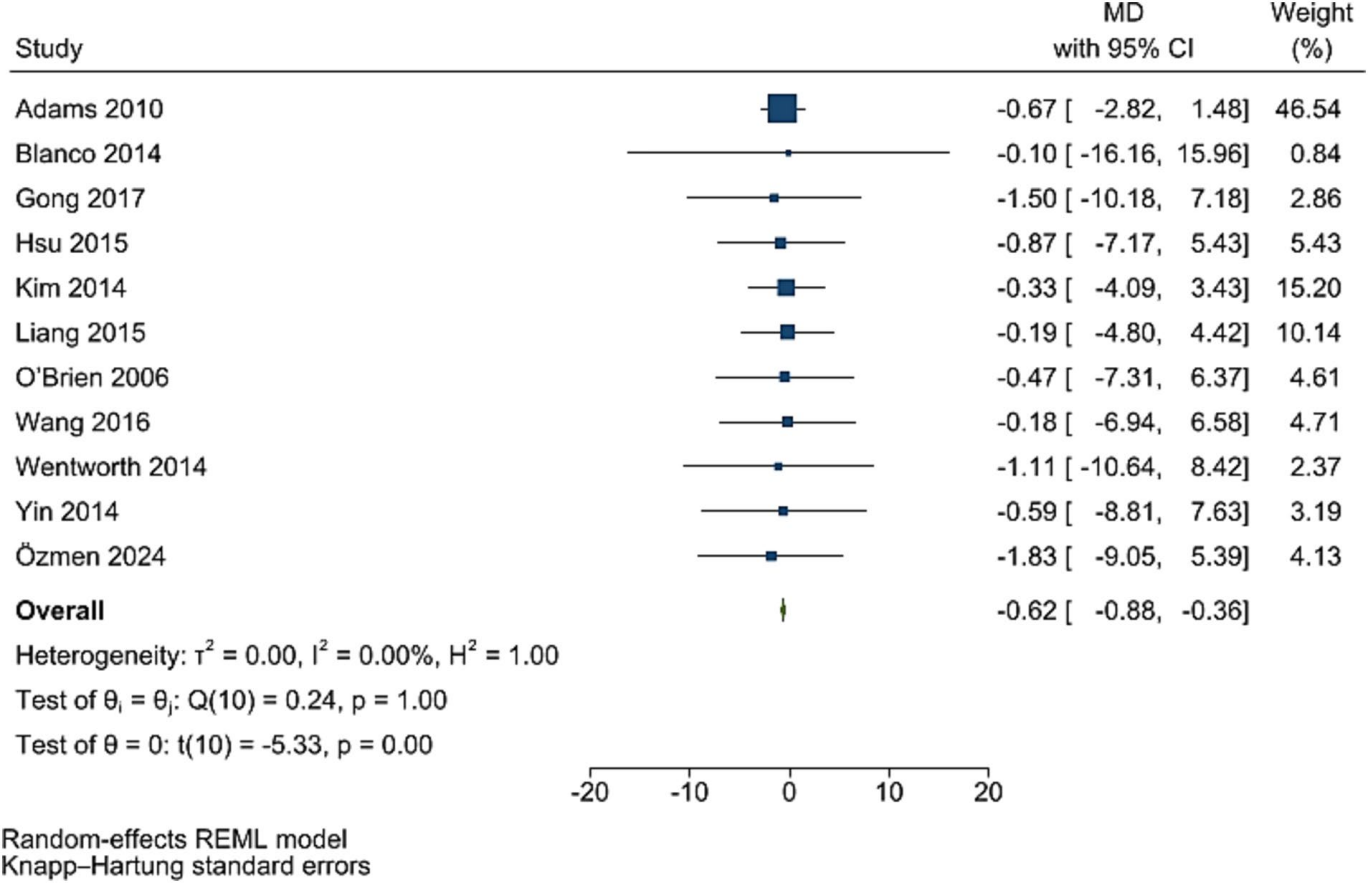
Forest plot detailing mean difference and 95% confidence intervals (CIs), the effects of bariatric surgery supplementation on insulin.

Post bariatric surgery, compared to pre-surgery values from 11 studies involving 1,831 participants, led to a significant reduction in insulin levels (WMD = −0.62, 95% CI: −0.88, −0.36, *p* < 0.001, *I*^2^ = 0.0%; tau^2^ value 0.001; Figure 7). A sensitivity analysis showed no significant change in effect overall estimates after the removal of single studies. Funnel plot was symmetrical (Supplementary Figure S7) with no significant Egger’s test (*p* = 0.922).

## Discussion

The findings from this meta-analysis underscore the profound impact of bariatric surgery on improving glycemic and metabolic outcomes in obese individuals with T2DM. Despite the overall improvements in glycemic parameters, the effect of bariatric surgery on HbA1c levels was not statistically significant in the primary analysis. This finding may reflect the complex interplay of factors influencing long-term glycemic control, including patient adherence, variability in surgical procedures, and baseline glycemic status. Notably, the GRADE quality for HbA1c was rated as low, indicating limited confidence in the reliability of this outcome. This suggests that future high-quality, long-term RCTs are needed to better clarify the true effect of bariatric surgery on HbA1c. Interestingly, after performing the trim and fill analysis to account for potential publication bias, the effect on HbA1c became statistically significant, highlighting how methodological limitations and potential reporting biases may obscure true effects. Significant reductions were observed in FBG, PPG, and HOMA-IR, along with modest improvements in C-peptide and fasting insulin levels. Evidence on FBG, PPG, C-peptide levels, HOMA-IR, and fasting insulin levels had high GRADE quality. These results align with the growing body of evidence demonstrating that bariatric surgery offers not only weight loss but also effective glycemic control and metabolic regulation, often resulting in partial or complete diabetes remission.

Multiple meta-analyses of RCTs have shown dramatic improvements in glycemic control and diabetes remission with bariatric surgery. For example, Wu et al. pooled 8 RCTs and found bariatric surgery gave a ~ 5.8-fold higher remission rate and much larger drops in HbA1c and fasting glucose than medical therapy (57). Kim et al. similarly reported T2DM remission rates of ~47% after RYGB, 42% after sleeve, and 25% after banding, versus only ~5% with medical care; mean HbA1c fell by ~0.96–0.97% after RYGB/SG but not with medical treatment (58). Meta-analyses comparing surgical procedures found RYGB tends to induce higher short-term remission than SG. Borgeraas et al. analyzed 10 RCTs and observed 1-year remission of 57% after RYGB vs. 47% after SG (RR ≈ 1.20), though by 2–5 years the rates were similar (7). Large pooled analyses (n ≈ 22,000) have also reported ~83.8% diabetes resolution after RYGB vs. ~ 47.8% after gastric banding (59). Overall, prior work consistently showed that more aggressive procedures (RYGB, BPD) yielded higher remission and glycemic improvements than restrictive ones. Few meta-reviews focused on insulin/HOMA. Rao et al. (2012) documented dramatic insulin-sensitivity gains after surgery – e.g. HOMA-IR fell by ~40–60% or more by 6–12 months, with particularly rapid drops after RYGB and BPD (60). However, most earlier meta-analyses did not report insulin, C-peptide, or HOMA-IR changes. These earlier analyses generally pooled relatively small numbers of trials and focused on a limited set of outcomes (mostly weight, HbA1c, fasting glucose, and remission). Many did not stratify results by procedure type or include newer RCTs, and none had fully comprehensive quality/GRADE appraisals of the evidence.

The significant reduction in FBG levels, as highlighted by the pooled WMD of −0.82 mg/dL, was consistent with previous studies, further emphasizing the effectiveness of surgical intervention over conventional medical management (61). The high heterogeneity observed in this analysis is consistent with variability in baseline characteristics, surgical techniques, and follow-up durations among the included studies. Following the trim-and-fill analysis, the pooled estimate for HbA1c demonstrated a WMD of −3.99%, confirming a substantial and clinically meaningful impact of bariatric surgery on long-term glycemic control compared to non-surgical interventions. This adjusted estimate suggests that the initial non-significant findings may have been influenced by publication bias. Our results align with prior long-term studies reporting sustained reductions in HbA1c levels up to 5 years postoperatively in patients undergoing bariatric surgery (61). While heterogeneity remains a concern, likely due to differences in patient populations and pre-surgical glycemic status, the findings strongly support the role of bariatric surgery as a metabolic intervention for diabetes management. The results for PPG reduction were consistent with prior studies that demonstrated improved incretin responses post-surgery, which contributes to better PPG control (62). The modest improvement in C-peptide levels, reflected by the pooled WMD of 0.38 ng/mL, suggests partial restoration of beta-cell function following bariatric surgery. This finding corroborates earlier research indicating that bariatric surgery improves pancreatic function and preserves residual beta-cell activity (63). However, the variability in results across studies points to heterogeneity in baseline beta-cell function and the degree of insulin resistance among patients. The significant reduction in HOMA-IR levels, with pooled WMD of −2.81, highlights the role of bariatric surgery in reducing insulin resistance, a hallmark of T2DM. Prior studies have consistently demonstrated that weight loss and improved adipokine profiles post-surgery contribute to enhanced insulin sensitivity (63). Lastly, the improvement in fasting insulin levels (pooled WMD of −0.62 μU/mL) aligns with prior evidence indicating that bariatric surgery reduces compensatory hyperinsulinemia. This outcome is likely driven by a combination of reduced insulin resistance and improved glucose metabolism post-surgery (64). The variability observed in the magnitude of fasting insulin changes across studies may reflect differences in baseline insulin levels, surgical methods, and follow-up durations. Overall, the results strongly support bariatric surgery as an effective intervention for improving glycemic control and metabolic outcomes in obese patients with T2DM.

The observed reductions in fasting blood glucose, postprandial glucose, and HbA1c levels post-bariatric surgery can be attributed to several physiological mechanisms (65). One of the most significant is the improvement in insulin sensitivity due to weight loss and changes in adipokine secretion. Bariatric surgery reduces visceral adiposity, which is strongly associated with insulin resistance, and leads to decreased secretion of pro-inflammatory cytokines like tumor necrosis factor-alpha (TNF-*α*) and interleukin-6 (IL-6). These changes reduce systemic inflammation and improve insulin signaling (66). Additionally, the alterations in gut hormone dynamics play a critical role. Increased levels of GLP-1 following surgery enhance glucose dependent insulin secretion, suppress glucagon release, and slow gastric emptying, all of which contribute to better glycemic control (67). The improvement in C-peptide levels and reductions in HOMA-IR suggest partial restoration of beta-cell function and reduced insulin resistance (68). The “foregut hypothesis” proposes that exclusion of the proximal small intestine enhances insulin sensitivity, while the “hindgut hypothesis” highlights the role of accelerated nutrient delivery to the distal intestine in stimulating GLP-1 secretion. Furthermore, bariatric surgery leads to a reduction in hepatic glucose output, likely through decreased lipotoxicity and improved liver insulin sensitivity. These metabolic changes collectively contribute to the observed reductions in fasting insulin levels and better overall glycemic control (69).

This meta-analysis is comprehensive, synthesizing data from 39 studies with a large sample size of participants, which enhances the robustness of the findings. The inclusion of multiple glycemic and metabolic outcomes provides a nuanced understanding of the impact of bariatric surgery on T2DM. Rigorous statistical methods, including sensitivity analyses and assessments of publication bias, lend credibility to the results. The adherence to PRISMA guidelines ensures transparency and reproducibility, making the findings reliable for clinical and research purposes. Despite its strengths, this review has several limitations. It is important to acknowledge that many included studies were rated as having a high risk of bias, primarily due to variability in laboratory follow-up protocols and inconsistent reporting standards. These factors may reduce the overall confidence in the pooled results and should be considered when interpreting the findings. The substantial heterogeneity observed across studies for outcomes like FBG, and PPG levels limits the generalizability of the findings. The included studies exhibited considerable variability in sample sizes and follow-up durations, ranging from as few as 6 to nearly 480 participants and from just over 2 months to 10 years. Such heterogeneity may influence the precision and generalizability of the pooled estimates. Smaller studies are more prone to random error, and shorter follow-up periods may not capture the durability of metabolic improvements, particularly for longer-term markers like HbA1c. This variation highlights the need for more standardized, long-term trials with adequate sample sizes to confirm and extend these findings. Additionally, the lack of uniform reporting on comorbid conditions and medication use makes it challenging to isolate the effects of bariatric surgery. Finally, factors such as the presence of cardiovascular disease, hypertension, or dyslipidemia, as well as the type and duration of antidiabetic medication use can significantly affect metabolic outcomes and glycemic response following bariatric surgery. Due to inconsistent or missing data, we were unable to perform adjusted analyses or stratify results based on these clinical variables. Future studies should prioritize standardized reporting of comorbidity profiles and medication regimens to allow for more precise assessments of surgical efficacy and its interaction with medical management.

The findings of this meta-analysis have significant implications for clinical practice. Bariatric surgery should be considered a key therapeutic option for obese patients with T2DM, particularly those with poor glycemic control despite optimal medical therapy. The observed reductions in fasting blood glucose, and postprandial blood glucose levels suggest that surgery can achieve better glycemic outcomes compared to conventional treatments. These benefits are likely to translate into reduced risks of diabetes-related complications, such as retinopathy, nephropathy, and cardiovascular disease. Additionally, the improvements in insulin sensitivity and beta-cell function may delay or prevent disease progression, further enhancing patient quality of life. Clinicians should weigh the risks and benefits of surgery and provide individualized recommendations based on patient preferences, comorbidities, and surgical eligibility criteria.

Future research should focus on understanding the long-term durability of glycemic and metabolic improvements following bariatric surgery. Studies with extended follow-up durations are needed to assess the sustainability of outcomes such as HbA1c and HOMA-IR reductions. Randomized controlled trials comparing different bariatric procedures in diverse populations could provide valuable insights into procedure-specific effects. Additionally, research should explore the role of genetic and epigenetic factors in modulating responses to surgery. Mechanistic studies investigating changes in gut microbiota and their relationship with glycemic outcomes could shed light on novel pathways of metabolic regulation. Finally, economic evaluations assessing cost-effectiveness in different healthcare settings could guide policy decisions.

## Conclusion

This meta-analysis demonstrates that bariatric surgery significantly improves glycemic and metabolic outcomes in obese individuals with T2DM. The substantial reductions in FBG, and PPG, coupled with improvements in insulin sensitivity and beta-cell function, highlight the metabolic benefits of surgical intervention beyond weight loss. These findings underscore the need for greater integration of bariatric surgery into diabetes care pathways, offering hope for better disease management and improved patient outcomes.

## Data Availability

The datasets presented in this study can be found in online repositories. The names of the repository/repositories and accession number(s) can be found in the article/Supplementary material.

## Footnotes

^1^ https://www.who.int/news-room/fact-sheets/detail/obesity-and-overweight

^2^ https://www.who.int/news-room/fact-sheets/detail/diabetes
