# Supplementary material for: Effect of bariatric surgery on glycemic and metabolic outcomes in people with obesity and type 2 diabetes mellitus: a systematic review, meta-analysis, and meta-evidence of 39 studies
Source: Front Nutr. 2025 Jun 23;12:1603670. doi: 10.3389/fnut.2025.1603670 (PMC12229798; doi:10.3389/fnut.2025.1603670)
Supplement: Supplementary file 1 [file Data_Sheet_1.docx]

**Search Strategy:**

Scopus:

(TITLE-ABS-KEY("Bariatric Surgery" OR "gastric bypass" OR "Roux-en-Y gastric bypass" OR "sleeve gastrectomy" OR "adjustable gastric banding" OR "biliopancreatic diversion" OR "laparoscopic surgery" OR "gastric band" OR "metabolic surgery" OR "weight loss surgery" OR "obesity surgery" OR "bariatric procedure" OR "obesity treatment" OR "surgical weight loss")) AND (TITLE-ABS-KEY("type 2 diabetes" OR "T2DM" OR "diabetes mellitus, type 2" OR "insulin resistance" OR "hyperglycemia" OR "blood glucose" OR "glycemic control" OR "HbA1c" OR "glycated hemoglobin" OR "fasting glucose" OR "postprandial glucose" OR "glucose metabolism" OR "beta cell function" OR "insulin secretion" OR "insulin sensitivity" OR "C-peptide" OR "HOMA-IR")) AND (TITLE-ABS-KEY("body mass index" OR "BMI" OR "obesity" OR "obese patients" OR "morbid obesity" OR "severe obesity" OR "overweight" OR "high BMI" OR "obesity management" OR "adiposity")) AND (TITLEABS- KEY("outcomes" OR "effectiveness" OR "impact" OR "remission" OR "long-term outcomes" OR "short-term outcomes" OR "diabetes remission" OR "weight loss outcomes" OR "glycemic outcomes" OR "glucose outcomes" OR "insulin outcomes" OR "metabolic outcomes"))

PubMed:

("Bariatric Surgery"[MeSH Terms] OR "gastric bypass"[MeSH Terms] OR "Roux-en-Y gastric bypass"[Title/Abstract] OR "sleeve gastrectomy"[Title/Abstract] OR "adjustable gastric banding"[Title/Abstract] OR "biliopancreatic diversion"[Title/Abstract] OR "laparoscopic surgery"[Title/Abstract] OR "gastric band"[Title/Abstract] OR "metabolic surgery"[Title/Abstract] OR "weight loss surgery"[Title/Abstract] OR "obesity surgery"[Title/Abstract] OR "bariatric procedure"[Title/Abstract] OR "obesity treatment"[Title/Abstract] OR "surgical weight loss"[Title/Abstract]) AND ("type 2 diabetes"[Title/Abstract] OR "T2DM"[Title/Abstract] OR "diabetes mellitus, type 2"[MeSH Terms] OR "insulin resistance"[Title/Abstract] OR "hyperglycemia"[Title/Abstract] OR "blood glucose"[MeSH Terms] OR "glycemic control"[Title/Abstract] OR "HbA1c"[Title/Abstract] OR "glycated hemoglobin"[Title/Abstract] OR "fasting glucose"[Title/Abstract] OR "postprandial glucose"[Title/Abstract] OR "glucose metabolism"[Title/Abstract] OR "beta cell function"[Title/Abstract] OR "insulin secretion"[Title/Abstract] OR "insulin sensitivity"[Title/Abstract] OR "Cpeptide"[ Title/Abstract] OR "HOMA-IR"[Title/Abstract]) AND ("body mass index"[MeSH Terms] OR "BMI"[Title/Abstract] OR "obesity"[MeSH Terms] OR "obese patients"[Title/Abstract] OR "morbid obesity"[Title/Abstract] OR "severe obesity"[Title/Abstract] OR "overweight"[Title/Abstract] OR "high BMI"[Title/Abstract] OR "obesity management"[Title/Abstract] OR "adiposity"[Title/Abstract]) AND ("outcomes"[Title/Abstract] OR "effectiveness"[Title/Abstract] OR "impact"[Title/Abstract]OR "remission"[Title/Abstract] OR "long-term outcomes"[Title/Abstract] OR "short-term outcomes"[Title/Abstract] OR "diabetes remission"[Title/Abstract] OR "weight loss outcomes"[Title/Abstract] OR "glycemic outcomes"[Title/Abstract] OR "glucose outcomes"[Title/Abstract] OR "insulin outcomes"[Title/Abstract] OR "metabolic outcomes"[Title/Abstract])


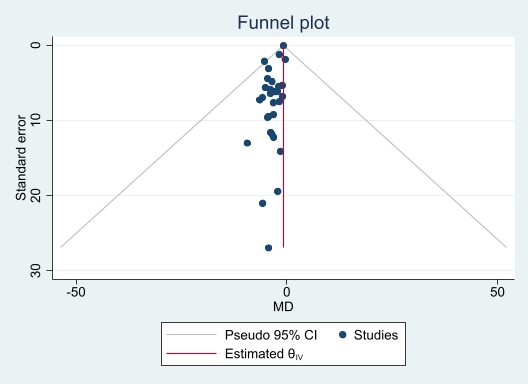


Figure.S1. Funnel plot for FPG.


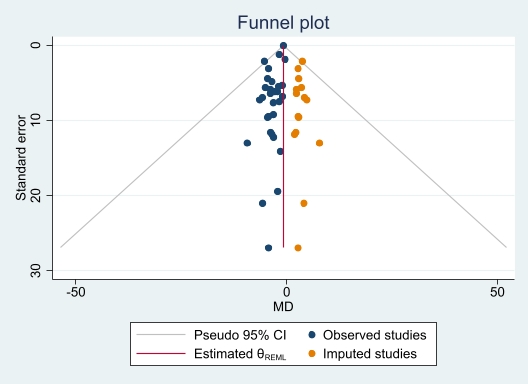


Figure.S2. Trim and filled for FPG.


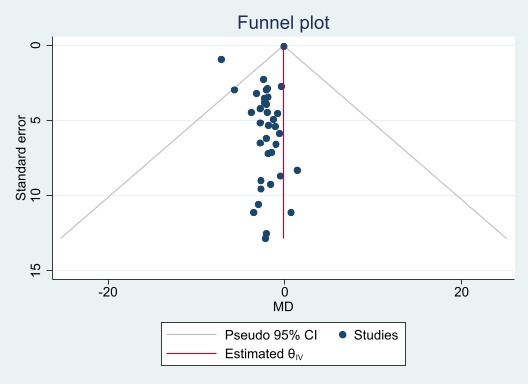


Figure.S3. Funnel plot for HbA1C.


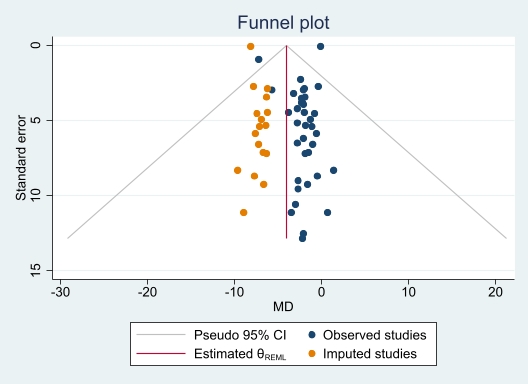


Figure.S4. Trim and filled for HbA1C.


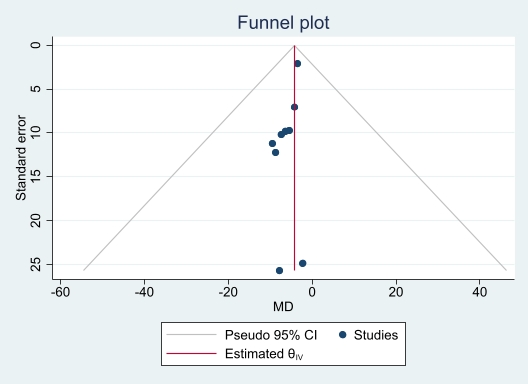


Figure.S5. Funnel plot for PPG.


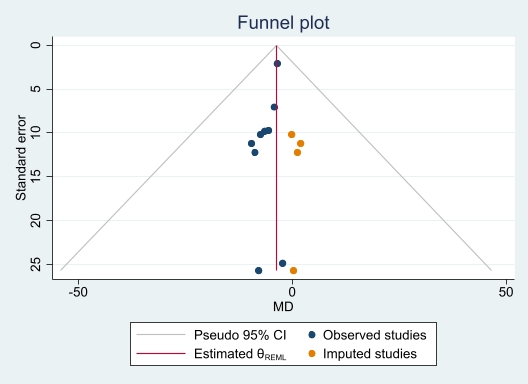


Figure.S6. Trim and filled for PPG.


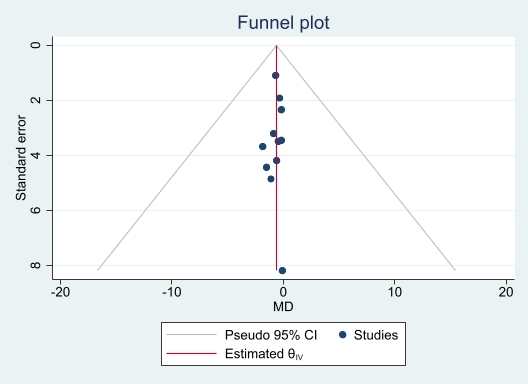


Figure.S7. Funnel plot for insulin.
